# Prognostic Impact of Direct ^131^I Therapy After Detection of Biochemical Recurrence in Intermediate or High-Risk Differentiated Thyroid Cancer: A Retrospective Cohort Study

**DOI:** 10.3389/fendo.2019.00737

**Published:** 2019-10-29

**Authors:** José F. Carrillo, Rafael Vázquez-Romo, Margarita C. Ramírez-Ortega, Liliana C. Carrillo, Edgar Gómez-Argumosa, Luis F. Oñate-Ocaña

**Affiliations:** ^1^Departmento de Cabeza y Cuello, Instituto Nacional de Cancerología, Mexico, Mexico; ^2^Subdirección de Cirugía, Instituto Nacional de Cancerología, Mexico, Mexico; ^3^Departmento de Farmacología, Instituto Nacional de Cardiología Ignacio Chávez, Mexico, Mexico; ^4^Departmento de Cuidados Paliativos, Instituto Nacional de Cancerología, Mexico, Mexico; ^5^Departmento de Medicina Nuclear, Instituto Nacional de Cancerología, Mexico, Mexico; ^6^Subdirección de Investigación Clínica, Instituto Nacional de Cancerología, Mexico, Mexico

**Keywords:** differentiated thyroid cancer, ^131^I treatment, thyroglobulin, diagnostic whole body scan, cohort studies

## Abstract

**Background:** Patients treated for intermediate- or high-risk differentiated thyroid carcinoma (DTC) and Thyroglobulin (TG) elevation during follow-up, require a diagnostic whole-body scan (DWBS) and if positive, ^131^I treatment. This approach can lead to a delay in treatment and increased costs. The purpose of this study is to compare the oncologic outcomes associated to administration of direct therapy with ^131^I at first biochemical recurrence.

**Methods:** Retrospective cohort study of patients with intermediate- or high-risk DTC treated with total thyroidectomy, ^131^I ablation and who developed TG elevation during follow-up, between January 2007 and December 2015. Cohort A included patients who underwent a DWBS with 5 mCi of ^131^I, and if negative an MRI and/or ^18^FDG PET-CT prior to the therapeutic dosage, and cohort B included those who only received a therapeutic dosage of ^131^I, without a DWBS or extensive image studies. Main outcomes were second recurrence (SR) and disease-free survival (DFS). The diagnostic accuracy of DWBS was analyzed.

**Results:** Cohorts A and B had 74 and 41 patients, each. By multivariate analysis, age, differentiation grade, TN classification, ablation dose, and performed DWBS (odds ratio 55.1; 95% CI 11.3–269) were associated with SR (*p* < 0.0001); age, male gender, ablation dose and performed DWBS (hazard ratio 7.79; 95% CI 3.67–16.5) were independent factors associated with DFS (*p* < 0.0001). DWBS diagnostic accuracy was 36.48%.

**Conclusion:** ^131^I treatment in patients with DTC biochemical recurrence and no DWBS or extensive image studies is associated with a significantly lower frequency of SR and an increased DFS. The diagnostic accuracy of DWBS is low, and its clinical efficiency should be defined in prospective phase III studies.

## Synopsis

Biochemical recurrences after treatment of intermediate- or high-risk thyroid cancer require a diagnostic whole-body scan and if positive, ^131^I treatment. This study suggests that patients could receive treatment without a scan, extensive image studies or long follow-up, to decrease recurrence rate and increase disease-free survival.

## Introduction

The management of patients with differentiated thyroid carcinoma (DTC) treated with thyroidectomy and ^131^I ablation and who during follow-up develop progressive elevation of serum Thyroglobulin (TG), is the administration of 5 milliCuries (mCi) of ^131^I to obtain an exploratory diagnostic whole-body scintigraphy scan (DWBS), CT (computed tomography), or MRI (magnetic resonance image), and even a ^18^FDG PET-CT or continued follow up of thyroglobulin levels. If image studies are positive, or TG levels continue to increase, patients receive an ^131^I therapeutic dose of 50–200 mCi (1, 2).

The diagnostic accuracy of a 5 mCi ^131^I DWBS in the presence of a detected or suspected biochemical recurrence has not been reported and has been insufficiently investigated in studies yielding mostly inconclusive results (3–6). This raises the possibility of greater false-negative rates than those previously reported, and the consequent impact on final outcomes. Patients with a progressive increase in TG or anti-TG antibodies levels –even when high-risk factors are present– and a negative DWBS, the so called TENIS syndrome (7), are continuously followed with periodic DWBS until clinical recurrence, a positive DWBS or a greater increase in TG or anti-TG antibodies occurs. Moreover, some studies suggest performance of a 99mTc-3PRGD2 SPECT/CT in these circumstances (8). This policy delays treatment, increases costs, and induces stunning in the recurrent thyroid tissue, with its possible deleterious impact on prognosis.

The administration of 150–200 mCi of ^131^I in the presence of a biochemical recurrence without a DWBS, aforementioned image studies or continuous follow-up, is not routinely dispensed due to the assumed low ^131^I uptake and the risk of secondary malignancies, bone marrow dysplasia, toxic secondary effects, and endocrine dysfunction (9, 10). Therefore, the uptake rate and the oncologic outcomes of this treatment strategy have not been reported, but indirect study conclusions on ^131^I administration when TG is elevated and the DWBS is negative (11).

We analyzed the prognostic impact of direct ^131^I therapeutic dose in DTC after biochemical recurrence, in a cohort of patients with intermediate- and high-risk DTC, in terms of the frequency of a second recurrence (SR) and disease-free survival (DFS). The diagnostic accuracy of the DWBS was also evaluated.

## Materials and Methods

### Patients

Retrospective cohort study of patients with intermediate- or high-risk DTC, treated at our Institution between January 2007 and December 2015. Inclusion criteria: patients older than 18 years with histopathology reports demonstrating DTC (papillary, follicular, or mixed papillary/follicular carcinoma). High-risk was defined as an age above 55 years and one or more of these factors: tumor size over 4 cm, extension to soft tissue, clinical lymph node involvement, or poor differentiation. Patients younger than 55 but with two or more of the aforementioned factors, were considered intermediate-risk (1, 2). Patients treated with total thyroidectomy followed by adjuvant ^131^I ablation (100–150 mCi) were included if, during follow-up, they developed a biochemical recurrence defined as a Tg elevation ≥2 ng/ml and/or anti-Tg antibodies ≥50 IU/ml, under Thyroid stimulating hormone (TSH) suppression (serum level <0.5 mIU/L), with no clinical or image evidence of disease (4, 10). Follow-up visits were scheduled every 4 months during the first 2 years after thyroidectomy, then twice a year, and annually afterwards. Neck ultrasound and chest x-ray were obtained every year, and when the TG value increased.

Cohort A were treated according to ATA guidelines. Patients underwent a DWBS with 5 mCi of ^131^I prior to the therapeutic dosage. If uptake was positive, they received a ^131^I dosage of 150–200 mCi. A head/neck and chest CT scan and/or magnetic resonance imaging (MRI) was performed if the DWBS was negative, as well as a ^18^FDG PET/CT since 2011. If the aforementioned tests were negative for metastases or recurrent disease, follow-up continued until the DWBS was positive or if TG levels continued to increase, and a therapeutic ^131^I dosage was administered. If clinical-image (structural) recurrences ensued, these were treated with surgery if feasible, and/or radiation therapy, followed by a ^131^I therapeutic dose.

Patients assigned to one of the authors' (JFC) outpatient clinic for follow-up after thyroidectomy and ablative ^131^I treatment represent cohort B. To avoid delay in management and according to reports which advice direct administration of ^131^I in case of increasing TG levels, these patients received a therapeutic dosage of ^131^I without a prior DWBS or extensive image studies, but if this scan was negative an MRI and/or an ^18^FDG PET/CT were performed -as in cohort A- to search for structural recurrences elsewhere.

There were no major differences regarding outpatient clinic visits or follow-up tests in cohorts A or B, except regarding diagnostic and therapeutic strategy when a biochemical recurrence occurs.

The study protocol is STROBE-compliant and was accepted by the IRB and Ethics committee (01/2017). Both committees have waived the requirement for written informed consent because of the retrospective nature of the study.

### Variables

Clinical, baseline and follow-up blood cytology and biochemistry, ultrasonography and x-ray data were recorded. Serum TG (ELSA-HTG immunoradiometric assay, CISBio International, Codolet, France), and anti-TG antibodies levels (Immulite 2000 Analyzer, Siemens Healthcare GmbH, Erlangen, Germany), as well as serum TSH (radioimmunometric assay, SPAC-5TSHkit, Daiichi, Japan) were measured. DWBS was performed after TSH stimulation after a 20–30 days withdrawal of levothyroxine administration. If TSH levels were ≥30 mIU/L, a 5 mCi ^131^I dosage was administered and the DWBS was obtained 48 h later (Symbia T6, Siemens Medical Solutions USA, Inc., Malvern, PA, USA). ^131^I treatment was conducted following the same parameters and with the equipment mentioned above; a dose of 150–200 mCi was administered and a scan was obtained 5–10 days later. Scan with planar images was performed in 100% of cases. SPECT-CT was performed in 47 cases (40.86%).

### Statistical Analysis

Sample size was calculated assuming a frequency of SR of 90% in cohort A and 70% in cohort B, with 80% power, a probability value of 0.05, and an allocation ratio of 2:1; a total of 132 patients was necessary (88 in cohort A and 44 in cohort B). Student's T or the Chi squared tests were used for comparisons. Factors associated with SR were tested in a logistic regression model (12). SR was defined as the event characterized by an increase in Tg (≥2 ng/ml), increased anti-TG antibodies (≥50 IU/ml) under TSH suppression (serum level ≤ 0.5 mIU/L), or a clinical or image recurrence detected by neck ultrasound, MRI of the neck and mediastinum or ^18^FDG PET/CT. Factors associated with DFS were tested with the Kaplan–Meier method, and the log-rank test was used for comparisons. DFS was calculated from the date of surgery until the SR event or the last visit. Multivariate analysis of prognostic factors associated with DFS was conducted with Cox's model (13). The diagnostic accuracy of DWBS in biochemical recurrences was determined (14). The standard for positivity in DWBS refers to clinical or imaging recurrence during follow-up, a positive follow-up DWBS, or persistent elevation of TG or anti-TG antibodies levels after DWBS, which made surgical resection imperative and/or the final administration of a therapeutic dosage of ^131^I. Probability values of 0.05 or lower were considered significant using two-tailed statistics. SPSS software for Mac, version 23 was used (IBM Corp., Armonk, NY, USA).

## Results

### Patients

One-hundred and fifteen patients were included, 85 women (73.9%) and 30 men (26.1%), with a mean age of 50.9 years (Standard deviation 15.7, range 19–93); 74 (64.3%) were allocated to cohort A and 41 (35.7%) to cohort B. Demographic and clinical characteristics are presented in Table 1. There were 105 papillary tumors, one Hürthle cell carcinoma, three insular carcinomas and six other histopathologic varieties (tall cell and poorly-differentiated carcinomas). No significant differences were detected in terms of gender, histology, tumor and node classification (TNM), original thyroid tumor size, median TG levels, and pre- or post-operative radiotherapy treatment between cohorts. TSH mean value was 0.2 mIU/L for cohort A, range 0.01–4.5 mIU/L, median 0.1 mIU/L. Cohort B values were: mean 0.1 mIU/L, range 0.005–2 mIU/L median: 0.8 mIU/L.

**Table 1:** Clinical characteristics of patients per cohort (*n* = 115).

**Factor**	**Cohort A**	**Cohort B**	***p***
	**(*n* = 74)**	**(*n* = 41)**	
**Age (years)**	52.9 (14.5)	47 (17)	**0.05**
Gender^*^			
Female	58 (68.2)	27 (31.8)	0.143
Male	16 (53.3)	14 (46.7)	
Subtype^*^			
Papillary	67 (64.4)	37 (35.6)	0.666
Hürthle cell	1 (100)	0	
Insular cell	2 (66.7)	1 (33.3)	
Other	4 (57.1)	3 (42.9)	
**Size (major diameter, cm)**	4.46 (2.34)	4.53 (2.17)	0.869
T (TNM)^*^			
T1 or T2	9 (56.3)	7 (43.8)	0.692
T3	39 (63.9)	22 (36.1)	
T4	26 (68.4)	12 (31.6)	
N (TNM)^*^			
N0	16 (61.5)	10 (38.5)	0.744
N1	58 (65.16)	31 (34.8)	
Pre-operative ExRT^*^	1 (50)	1 (50)	0.669
Post-operative ExRT^*^	3 (75)	1 (25)	0.651
**Ablation** ^**131**^**I dose (mCi)**	137 (35.2)	153 (38.8)	**0.027**
**First treatment** ^**131**^**I dose (mCi)**	158.1 (35.1)	171.9 (31.7)	**0.039**
Biochemical recurrence^*^			
No	6 (15.8)	32 (84.2)	
Yes	68 (88.3)	9 (11.7)	**<0.0001**
Clinical recurrence^*^			
No	48 (59.3)	33 (40.7)	0.079
Yes	26 (76.5)	8 (23.5)	

TNM, tumor-node-metastases; ExRT, external radiotherapy; mCi, millicurie; TSH, thyroid stimulating hormone; numbers represent means (standard deviation);

* *represent categorical variables and numbers represent the number of patients (percentage); p, probability values and bold numbers represent significant values*.

Cohort A median Tg was 4 ng/ml (minimum 0.2, maximum 6.4); Cohort B, median Tg was 2.5 ng/ml (minimum 0.1; maximum 4.96). Median anti-TG antibodies level was 60 IU/ml (range 55–5,450 IU/ml) at time of recurrence.

Transient recurrent laryngeal nerve lesion (RLNL) and hypoparathyroidism were recorded in 16 (13.9%) and 34 (29.6%) cases, respectively. At 1-year follow-up, permanent hypoparathyroidism had developed in 15 (13%) cases and RLNL in 10 (8.7%) cases. In cohort A, the DWBS was positive in 22 (29.7%) patients.

Positive uptake after therapeutic ^131^I dosage was 69 (93.2%) and 40 (97.6%) in cohorts A and B, respectively (*p* = 0.332).

Sites of uptake with planar images were located at: locoregional level, mediastinum, and lung in 40 (34.78%), 29 (25.2%), and 39 (33.91%) patients, respectively. One case had bone uptake and in 6 (5.21%) cases no uptake was detected.

DWBS sensitivity, specificity, negative and positive predictive values were 31, 100, 9.6, and 100% respectively. Diagnostic accuracy was 36.4%.

### Recurrences

SR during follow-up and after treatment of a first recurrence, was identified in 77 patients (67%). In cohort A, 68 patients (93.2%) presented a SR and in cohort B, 9 patients (22%) (*p* < 0.0001). The odds ratio was 3.85 (95% CI 2.15–6.89) (*p* < 0.0001) and the necessary number to treat was 1.55 (95% CI 1.28–1.99), both favoring cohort B. There were 34 patients (29.6%) with clinical recurrences during follow-up; 26 in cohort A (35.1%) and eight in cohort B (19.5%) (*p* = 0.061).

In cohort A, from 22 cases who had a positive DWBS, four (18.18%) patients finally developed a clinical recurrence which was treated with surgery in three cases-a neck dissection was performed because of neck recurrence-, and one with radiotherapy because of bone metastases.

Two patients received radiotherapy -in each cohort- because of unresectable disease considered by the treating surgeon. Afterwards all of them were subjected to an R0 resection. Three patients received adjuvant radiotherapy because of concerns on microscopic disease because of the locoregional extent of the malignancy.

Table 2 describes the bivariate association of relevant factors and the SR outcome. Risk factors significantly associated to SR by bivariate analysis were age, ^131^I ablation dose, first ^131^I therapeutic dose and exposure to DWBS. Risk factors associated with SR by multivariate analysis were age, non-papillary histology subtype, T and N classification, first ^131^I ablative dose, and exposure to DWBS (Table 3).

**Table 2:** Bivariate association of clinical factors with secondary biochemical recurrence (*n* = 115).

**Factor**	**OR (95% CI)**	***p***
**Cohort A (DWBS)**	22.39 (8.26–60.7)	**<0.0001**
**Cohort B (no DWBS)**	1	–
**Age (by years)**	1.05 (1.02–1.08)	**0.001**
**Age**		
<55 years	1	–
≥55 years	3.895 (1.59–9.55)	**0.003**
**Gender**		
Female	1	–
Male	0.737 (0.312–1.74)	0.486
**Subtype**		
Papillary	1	–
Other	2.59 (0.53–12.6	0.239
**Size (greater diameter, cm)**	0.957 (0.81–1.13)	0.603
**Thyroglobulin (first recurrence)**	1.0001 (0.997–1.004)	0.807
**T classification (TNM)**		
T1 or T2	1	0.215
T3	0.864 (0.278–2.68)	0.8
T4	1.933 (0.549–6.8)	0.305
**N classification (TNM)**		
N0	1	–
N1	1.87 (0.766–4.55)	0.17
**Extracapsular invasion**	1.33 (0.535–3.32)	0.537
**Pre-operative ExRT**	0.527 (0.032–8.66)	0.654
**Post-operative ExRT**	1.625 (0.163–16.15)	0.679
**Ablation** ^**131**^**I dose (mCi)**	0.984 (0.973–0.995)	**0.004**
**First therapeutic** ^**131**^**I dose (mCi)**	0.987 (0.976–0.999)	**0.034**

*DWBS, diagnostic whole-body scan; cm, centimeter; mCi, millicurie; TNM, tumor-node-metastases; ExRT, external radiotherapy; OR, odds ratio; CI, confidence interval; p, probability value and bold numbers represent significant values*.

**Table 3:** Multivariate association of clinical factors with second biochemical recurrence (*n* = 115).

**Factor**	**β (SE)**	**Exp β**	**95% CI**	***p***
**Cohort A**	4.01 (0.809)	55.1	11.286–269.3	**<0.0001**
**Cohort B**	–	1	–	
**Age (years)**	0.079 (0.025)	1.082	1.03–1.137	**0.002**
**Ablation** ^**131**^**I Dose (mCi)**	−0.034 (0.011)	0.966	0.946–0.988	**0.002**
**T classification (TNM)**				
T1 or T2	–	1	–	**0.04**
T3	−1.179 (1.058)	0.308	0.039–2.445	0.265
T4	1.02 (1.215)	2.773	0.256–29.97	0.401
**N classification (TNM)**				
N0	–	1	–	–
N1	1.93 (0.854)	6.869	1.268–36.63	**0.024**
**Subtype**				
Papillary	–	1	–	–
Other	3.743 (1.28)	42.23	3.435–519.1	**0.003**
**Constant**	−1.934 (1.75)	0.145	–	0.269

*TNM, tumor-node-metastases; mCi, millicurie; β, beta estimator; SE, standard error of beta estimator; Exp β, beta exponential (odds ratio); CI, confidence interval; p, probability values and bold numbers represent significant values. Model summary: −2 Log likelihood 67.32; Nagelkerke R Square 0.699; model p < 0.0001*.

### Survival

Median follow-up of patients in both cohorts was 3.2 years (interquartile range 1.9–5.7). Disease-specific (DSS) and Overall survival (OS) were not calculated because only two patients (2%) in cohort A and one (2.4%) in cohort B have died due to DTC (*p* = 0.532). The median DFS of both cohorts was 4.66 years (95% CI 3.2–6.04).

**Figure 1:**
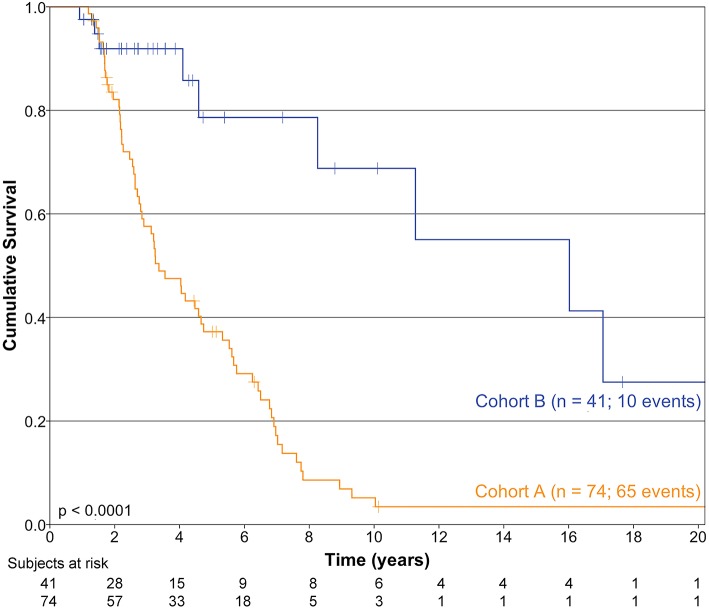
Kaplan-Meier curves depicting disease-free survival in cohorts A and B.

Median DFS for cohorts A and B were 3.36 (95% CI 2.42–4.3) and 16.02 years (95% CI 4.7–27.4), respectively (*p* < 0.0001). Kaplan-Meier DFS curves of cohort A and B are depicted in Figure 1.

The association of relevant factors and DFS by bivariate and multivariate analyses is described in Tables 4, 5, respectively.

**Table 4:** Bivariate association of clinical factors with disease-free survival (*n* = 115).

**Factor**	**HR (95% CI)**	***p***
**Cohort A (DWBS)**	4.284 (2.153–8.52)	**<0.0001**
**Cohort B (no DWBS)**	1	–
**Age (by years)**	0.993 (0.979–1.007)	0.332
**Age**		
<55 years	1	–
≥55 years	1.096 (0.69–1.741)	0.697
**Gender**		
Female	1	–
Male	1.463 (0.853–2.509)	0.167
**Subtype**		
Papillary	1	–
Other	2.007 (0.993–4.05)	0.052
**Size (greater diameter, cm)**	1.023 (0.9–1.163)	0.724
**Thyroglobulin (first recurrence)**	0.999 (0.998–1.001)	0.436
**T classification (TNM)**		
T1 or T2	1	0.165
T3	1.276 (0.609–2.673)	0.519
T4	1.864 (0.878–3.956)	0.105
**N classification (TNM)**		
N0	1	–
N1	1.739 (0.968–3.122)	0.064
**Extracapsular invasion**	1.479 (0.823–2.656)	0.19
**Pre-operative ExRT**	1.812 (0.248–13.22)	0.558
**Post-operative ExRT**	1.957 (0.605–6.327)	0.262
**Ablation** ^**131**^**I dose (mCi)**	1.007 (1.001–1.013)	**0.027**
**First therapeutic** ^**131**^**I dose (mCi)**	1.002 (0.995–1.01)	0.518

*DWBS, diagnostic whole-body scan; cm, centimeter; TNM, tumor-node-metastases; mCi, millicurie; ExRT, external radiotherapy; HR, hazards ratio; CI, confidence interval; p, probability value. Bold numbers represent statistical significance*.

**Table 5:** Multivariate association of clinical factors with disease-free survival (*n* = 115).

**Factor**	**β (SE)**	**Exp β**	**95% CI**	***p***
**Cohort A**	2.053 (0.384)	7.792	3.67–16.55	**<0.0001**
**Cohort B**	–	1	–	–
**Age (years)**	−0.018 (0.009)	0.982	0.966–0.999	**0.035**
**Gender**				
Female	–	1	–	–
Male	1.014 (0.302)	2.757	1.431–4.888	**0.002**
**Ablation** ^**131**^**I Dose (mCi)**	0.01 (0.003)	1.01	1.004–1.017	**0.002**

*mCi, millicurie; β, beta estimator; SE, standard error of beta estimator; Exp β, beta exponential (hazard ratio); CI, confidence interval; p, probability values and bold numbers represent significant values. Model summary: −2 Log likelihood 518.5; model p < 0.0001*.

## Discussion

The incidence of DTC and far advanced cases has increased, especially in low-income societies with access to specialized care, yielding intermediate- or high-risk cohorts (15–17).

Total thyroidectomy followed by adjuvant ^131^I ablation is the current treatment of DTC (18). However, long-term cancer control rates between doses have led to conflicting results. In low-risk cases, results of ablative therapy with ^131^I are probably equivalent between doses of 30–50 mCi and doses ≥100 mCi (19). In intermediate- and high-risk patients, higher-doses of ^131^I might produce better cancer control (20, 21).

There are reports of patients with no clinical or imaging recurrences in spite of persistently elevated TG or anti-TG antibodies, and in the presence of a negative DWBS during follow-up. In some cases, elevated marker levels (5–10%) decrease or disappear, and several authors analyze outcomes with the administration of “^131^I empirical therapy” at dosages ≥100 mCi, and lag spans up to 4 years (22, 23).

A cohort study of patients with DTC and long-term follow-up from a single cancer center with homogeneous surgical and radioiodine treatment protocols is presented. Cohort A received ^131^I treatment after confirmatory DWBS, Head and neck CT or MRI. From 2011 a ^18^FDG PET-CT was performed in these cases-which demonstrated a recurrence- or persistent elevation and increase of TG levels which prompted administration of ^131^I therapeutic dose. Cohort B received ^131^I treatment but no DWBS or further diagnostic studies or follow-up were performed.

An intermediate- to high-risk category was established in our patients because ≥80% had a T3 or above classification; their mean age was 51 years, lymph nodes were positive in >75% of cases, mean tumor size was 4.5 cm, major extrathyroidal extension was present in >90% of cases, and a small number of patients had aggressive histopathology (1, 24).

Mazzaferri reported the limited value of DWBS because of high false-negative rates and recommended a therapeutic dose of ^131^I with increasing TG values, in the presence of a negative neck ultrasound (6). However, he considered the detection of recombinant human TSH (rhTSH)-stimulated TG ≥2 ng/ml as indicative of treatment and this policy was used even in low-risk patients. This is controversial because of the slow progression of DTC and the high-cost of rhTSH (22). Others have considered the administration of an ^131^I dose when TG increases during follow-up, because of a high DWBS false-negative rate (67%), although they do not elaborate any further (6, 25, 26).

We address these controversies establishing a high efficiency strategy in the presence of biochemical recurrence, consisting in the direct administration of a therapeutic ^131^I dosage in high-risk patients. The administration in intermediate- and high-risk cases of a therapeutic ^131^I dose ≥100 mCi without the performance of a DWBS, follow up or extensive image studies, in the presence of a biochemical recurrence, was associated with a decrease in SR and increased DFS (4, 6).

Although we agree with recommendation 67B from ATA guidelines, sections (i) and (ii) regarding DWBS in patients with abnormal uptake outside the thyroid bed and large thyroid remnants on post-therapy WBS (1), these two situations are probably indicative of metastatic or residual disease and not necessarily represent biochemical recurrence, which is the subject of our study. Regarding section (iii) of same recommendation, anti-TG antibodies elevation in our series was successfully treated with direct ^131^I therapy in six cases. Two other cases belonged to cohort A and finally developed clinical recurrence which was treated surgically. The ^123^I isotope is not currently available in our country and its high-cost hampers its widespread use.

We agree with recommendation 68B from ATA guidelines which states that ^18^FDG PET-CT scan should be considered in high-risk patients with negative radioiodine scan, and this was included recently-as established in the manuscript- in the diagnostic strategy of cohort A. Even in cohort B cases if a negative uptake ensued after a direct ^131^I dose, we performed an MRI and ^18^FDG PET-CT of the mediastinum and neck from year 2011. However, a word of caution should be given because the sensitivity and specificity of this tool have been reported as 60 and 80% (27) in patients with stimulated TG ≥30 ng/ml. Aside, a major limit for its diagnostic accuracy is that significant findings are related to a ≥5 mm diameter of the lesion. Moreover, larger lesions could be considered structural recurrences which could be detected with CT and or MRI of head and neck and chest areas. Although our analyses was not designed to evaluate the diagnostic accuracy of ^18^FDG PET-CT diagnostic accuracy, sensitivity and specificity were 30.77, 25, and 100%, respectively, in those patients who had this test performed in our study, regarding biochemical recurrences.

The differentiation grade was associated with SR by multivariate analysis as has been reported (21, 24), and underscores the need to effectively treat patients with biochemical recurrence and high-risk factors, and avoid a DWBS (6). Although, as stated by ATA guidelines, recommendations for ^18^FDG PET-CT should be considered in cases with aggressive histology and metastatic disease to identify lesions at risk for rapid progression and evaluate the response after local or systemic therapy for invasive and metastatic disease, same aforementioned ^18^FDG PET-CT limitations regarding diagnostic accuracy of the test should be considered regarding this issue.

There is a tendency to administer lower ablation and therapeutic doses of ^131^I in DTC patients, even in intermediate-risk cases (1). In our study, higher ablative ^131^I doses were associated with decreased SR by multivariate analysis; this underscores the need to define the treatment strategy according to the clinical-histopathological characteristics and risks, especially in advanced stages (21).

RLNL and permanent hypoparathyroidism rates were high. Of note, these cases had more advanced disease and a high surgical risk (28, 29).

As in any cohort study, a potential pitfall in this study is the non-random and open nature of patient allocation in each cohort. The actual sample size is adequate because the observed differences between cohorts were greater than expected.

A clear difference exists in the uptake rate of DWBS compared with that after therapeutic doses in the setting of biochemical recurrences; the diagnostic accuracy of DWBS was 36.48%. This was analyzed in spite of the lack of a histopathological gold standard but the follow-up and TG levels are sufficient to establish the diagnostic accuracy values for DWBS (6, 26, 30). Moreover, no explanation exists for the positive ^131^I uptake after a direct therapeutic dose, after a 1-year follow-up with negative TG levels.

There are reports on the so-called “empiric” ^131^I dose administration after a negative DWBS, the TENIS syndrome (7), in patients who maintained persistent or elevated values of biochemical markers which made the administration of ^131^I therapy imperative or until clinical or image recurrence ensued (4, 10). These reports refer a high ^131^I uptake rate (≥65%) in the post-therapy scans. However, major drawbacks include the length of time before a therapeutic decision is made, stunning after repeated DWBS, and the high cost and delay of treatment because of the usually performed image studies (CT and ^18^FDG PET-CT scans). Also, over 50% of patients were low-risk and early-stage in these series, which decreases the clinical impact of this strategy (4, 10). In our study, over 95% of cases who received ^131^I therapy had a positive uptake.

Shinohara categorized TG and DWBS. Two groups with negative DWBS were described, one with TG elevation and another with normal TG (21). The first group had higher risk factors and a lower DFS. This suggests the need to take biochemical recurrences into consideration in high-risk patients and use a different strategy, perhaps a direct therapeutic dosage, as we have proposed.

We agree with some authors (1, 10) who consider that a biochemical incomplete response is not uncommon, with final return to normal limits in a majority of cases. Although a special consideration should be given to the fact that this regression refers mostly (10) to stimulated TG levels in low risk patients. Our study refers to a group of patients selected as intermediate- and high-risk with more stringent criteria for risk stratification, and to a non-stimulated cutoff of 2 ng/ml which is highly suspicious for a recurrence as demonstrated in our study. In intermediate- and high-risk cases -as established in our study-, spontaneous remission of high TG levels occurred in only 5 cases (6.75%) from patients who had a DWBS, with progressive increase of TG levels in the rest of them.

We agree with the ATA guidelines recommendations 80, 81, and 82 (1), regarding empiric radioiodine therapy in patients with increasing TG and anti-TG levels, although our success rate was higher (probably because of the inclusion in our series of high- and intermediate-risk patients), with 97% positive uptake rate and second recurrence rate of 21% after direct ^131^I therapy. A word of caution should be given considering the low sensitivity and negative predictive value of ^18^FDG-PETCT already mentioned.

With direct ^131^I therapy, the possibility of developing clinical or imaging metastases is decreased, since our strategy has the potential to prevent the appearance of resistant cell strains and therefore avoid stunning—which when unresectable, are frequently reported as resistant to radiotherapy (31, 32). Although there is evidence for the use of multitargeted kinase inhibitors in ^131^I-resistant cases, resistance and the need to administer these agents should be avoided (31).

There are reports of acute myeloid leukemia in patients with DTC treated with radioiodine (9). However, the total dosages used are usually not described. Furthermore, as stated by the same authors, the recurrence risk if no ^131^I therapy is used, is higher than the possibility of developing leukemia in high-risk cases.

In spite of a good prognosis in low-risk patients, some do recur, and early detection is imperative. The administration of ^131^I therapy in these patients should be judicious because of quality of life concerns and the risk of secondary malignancies (33). Lower doses in cases of a suppressed TG increase, could be indicated and investigated in the future, given our results.

A greater number of intermediate- and high-risk cases should be recruited to establish the approach reported as standard in the treatment of biochemical recurrences. More sensitive iodine scanners and studies proposing the rational use of ^18^FDG-PETCT might confirm our results. Prospective and multi-institutional studies to confirm these findings with possible impact on survival are warranted (8, 34, 35).

Intermediate- and high-risk DTC patients with TG elevation during follow-up should probably receive a therapeutic dose of ^131^I without a prior DWBS to prevent TENIS syndrome, treatment delays and increased costs. Other image studies like CT and MRI as well as ^18^FDG-PETCT and follow up of TG levels could be avoided. This approach improves DFS in patients with biochemical recurrence. DWBS diagnostic accuracy is low and its performance could induce stunning, delay treatment and increase clinical recurrence rates.

## Data Availability Statement

All datasets generated for this study are included in the article/supplementary material.

## Ethics Statement

The studies involving human participants were reviewed and approved by Comité de Investigación y Comité de Etica en Investigación, Instituto Nacional de Cancerología, México. Written informed consent for participation was not required for this study in accordance with the national legislation and the institutional requirements.

## Author's Note

This study was presented in part at the ENT World Congress (oral presentation), IFOS, Paris, June 24–28, 2017 (abstract:3665).

## Author Contributions

JC and LO-O: study concepts and literature review. JC, RV-R, MR-O, LC, EG-A, and LO-O: protocol writing. JC, RV-R, LC, and MR-O: data acquisition. JC, RV-R, MR-O, LC, EG-A, and LO-O: quality control of database. JC and LO-O: data analysis and interpretation. JC, RV-R, EG-A, and LO-O: manuscript drafting. JC, RV-R, MR-O, LC, EG-A, and LO-O: manuscript final review.

### Conflict of Interest

The authors declare that the research was conducted in the absence of any commercial or financial relationships that could be construed as a potential conflict of interest.
